# Forging the path to precision medicine in Qatar: a public health perspective on pharmacogenomics initiatives

**DOI:** 10.3389/fpubh.2024.1364221

**Published:** 2024-03-14

**Authors:** Kholoud Bastaki, Dinesh Velayutham, Areeba Irfan, Mohd Adnan, Sawsan Mohammed, Hamdi Mbarek, M. Waild Qoronfleh, Puthen Veettil Jithesh

**Affiliations:** ^1^Clinical and Pharmacy Practice Department, College of Pharmacy, QU Health, Qatar University, Doha, Qatar; ^2^College of Health & Life Sciences, Hamad Bin Khalifa University, Education City, Doha, Qatar; ^3^College of Medicine, Pre-Clinical Education Department, QU Health, Qatar University, Doha, Qatar; ^4^Qatar Precision Health Institute, Doha, Qatar; ^5^Q3 Research Institute (QRI), Research & Policy Division, Ann Arbor, MI, United States

**Keywords:** pharmacogenomics (PGx), population health, precision health, precision medicine, Middle East, Qatar

## Abstract

Pharmacogenomics (PGx) is an important component of precision medicine that promises tailored treatment approaches based on an individual’s genetic information. Exploring the initiatives in research that help to integrate PGx test into clinical setting, identifying the potential barriers and challenges as well as planning the future directions, are all important for fruitful PGx implementation in any population. Qatar serves as an exemplar case study for the Middle East, having a small native population compared to a diverse immigrant population, advanced healthcare system, national genome program, and several educational initiatives on PGx and precision medicine. This paper attempts to outline the current state of PGx research and implementation in Qatar within the global context, emphasizing ongoing initiatives and educational efforts. The inclusion of PGx in university curricula and healthcare provider training, alongside precision medicine conferences, showcase Qatar’s commitment to advancing this field. However, challenges persist, including the requirement for population specific implementation strategies, complex genetic data interpretation, lack of standardization, and limited awareness. The review suggests policy development for future directions in continued research investment, conducting clinical trials for the feasibility of PGx implementation, ethical considerations, technological advancements, and global collaborations to overcome these barriers.

## Introduction

Pharmacogenomics (PGx) is a rapidly growing field of medicine that focuses on the relationship between an individual’s genetic makeup and their response to medications. This field is a prominent example of precision medicine, as it enables the prediction of medication effectiveness, potential toxicity, and the most appropriate dosage (1, 2). The utilization of PGx testing in clinical setting has the potential to significantly transform healthcare delivery. By incorporating genetic information to tailor treatment plans, healthcare providers can improve patient outcomes and lower healthcare costs (3, 4). However, the implementation of pharmacogenomics in clinical practice has been a slow process. Historically, there have been challenges associated with the interpretation of genetic data and the integration of this information into clinical decision-making (2, 5).

The primary objective of this paper is to shed light on the critical role of PGx in the field of precision medicine and its potential impact on healthcare delivery. Specifically, we aim to explore the current state of PGx research and implementation in Qatar and its relevance in the global context. Qatar, with its rapidly advancing healthcare infrastructure and diverse population, serves as an ideal case study for understanding the challenges and opportunities associated with integrating PGx into clinical practice in the Middle East (6). By focusing on Qatar, we hope to provide insights that can be valuable not only to the local healthcare system but also to the broader international community interested in the advancements of PGx.

First, we will provide an overview of the current landscape of PGx in Qatar in the wider global context, emphasizing the ongoing research initiatives. Recognizing that education and awareness are pivotal for realizing PGx’s full potential in healthcare, we will then emphasize the value of educational efforts and other initiatives in Qatar that support the implementation of PGx. Finally, we will address challenges associated with implementation and outline future directions for the field.

## PGx research

### The global context

Globally, research has helped in identifying several genetic variants that are associated with response and potential adverse effects to various drugs (1, 7). Often such research involves a small number of patients from a specific population and hence generalizability and statistical significance are not achieved. At times, results from multiple studies are discordant for the same drug-gene pairs and unable to provide conclusive evidence in support of involvement of the genetic variant in drug response. Despite these issues, systematic efforts in mining the literature have identified a number of genes with enough evidence to be implicated in response.

For example, the pharmacogenomics knowledgebase (PharmGKB) has extensively curated information about the impact of genetic variation on drug response. Such information is organized under various categories, such as prescribing information, drug label annotations, curated pathways, clinical annotations and variant annotations (8, 9). While the variant annotation section provides information on the association between a genetic variant and a medication from a single publication, the clinical annotations section summarizes all such published evidence. The provision of a score and assigning a level of evidence to each genetic variant-drug combination based on all the evidence help in prioritizing which genes should be tested as priority. The sections on drug label annotations and prescribing information further help in understanding the practical requirements of genetic testing and how the test results should be interpreted.

Drug regulatory agencies in several countries specify drug labels. Moreover, agencies including the US Food and Drug Administration (FDA), Swissmedic, Japanese Pharmaceutical and Medical Devices Agency (PMDA) and the European Medicines Agency (EMA) in their labels provide information on genetic variants affecting the medication and also at times on the requirement of genetic testing prior to prescription of the drug (10, 11). Further support for the interpretation of PGx test results and how to incorporate these in the electronic medical records system are provided through guidelines by efforts such as the US-based Clinical Pharmacogenetic Implementation Consortium (CPIC), the Dutch Pharmacogenetics Working Group (DPWG) founded by the Royal Dutch Pharmacists Association (KNMP) in the Netherlands, Canadian Pharmacogenomics Network for Drug Safety (CPNDS) and the French National Network of Pharmacogenetics (RNPGx) (12). The CPIC has developed guidelines close to 100 genes – drugs pair (13). Similarly, the DPWG has so far developed 86 evidence-based gene-drug pair guidelines, of which close to 47 guidelines provide therapeutic recommendations for aberrant phenotypes that have been fully integrated into the electronic health records for clinical decision support (14, 15).

Several large studies are currently underway in different parts of the world to evaluate the use of gene panel based PGx approaches. Examples of studies from the USA include CLIPMERGE PGx, eMERGE-PGx, PG4KDS, IGNITE, INGENIOUS PGx, RIGHT 10 K, and the 1,200 Patients Project (16–22). For example, the “RIGHT 10 K” study is a collaborative effort between Mayo Clinic and Baylor College of Medicine, aimed at utilizing genomic data to personalize medical treatment for patients. The primary goal was to integrate preemptive, sequence-based PGx into routine clinical care practices. This involved proactively utilizing genetic information to guide drug prescribing decisions for patients. This study involves more than 10,000 patients at Mayo Clinic, who have had their pharmacogenes sequenced using samples from the Mayo Clinic Biobank. The study worked on developing the necessary tools and resources required to implement clinical pharmacogenomics effectively, including the creation of databases, algorithms, and reporting systems. Additionally, it conducted assessments to determine the prevalence of both well-documented common genetic variations, for which clinical guidelines are already established, and rare genetic variations that could be identified through DNA sequencing as opposed to traditional genotyping methods (23). In Europe, recently a randomized controlled trial (RCT) (PREPARE) was conducted in 8,100 patients as part of the U-PGx project to investigate the impact of pre-emptive PGx testing of a panel of 13 pharmacogenes. The study showed the viability and advantages of applying PGx decision assistance in a variety of European healthcare settings, including better prescribing practices and patient outcomes (24, 25). PGx research in Africa is also gaining traction, with a focus on addressing health disparities and improving drug access. The Human Heredity and Health in Africa (H3Africa) initiative is a prime example, involving multiple African countries. The African Pharmacogenomics Consortium (APC) was launched in 2018 to initiate PGx characterization of African diverse population when Caucasian and Asian population-based algorithms failed for the African population (26).

PGx diversity was assessed among 64 countries across Asia from the GenomeAsia 100 K Project pilot using a whole-genome sequencing dataset of 1,739 individuals of 219 population groups. This study predicted adverse drug reactions (ADRs) due to carbamazepine, clopidogrel, peginterferon and warfarin to vary between populations widely from zero to hundred percent (27). The Southeast Asian Pharmacogenomics Research Network (SEAPharm) was formed to enhance the understanding of PGx specifically in the region (28). The IndiGen national genome sequencing initiative in India analyzed 1,029 whole genomes and unveiled key PGx variants in Indians. Findings indicate notable disparities in clinically actionable PGx variant frequencies compared to global populations. A total of 134 common, potentially deleterious PGx variants affecting 102 pharmacogenes were identified. On average, each Indian individual carried eight PGx variants that can directly impact treatment choices or drug dosing (29). Findings from further analysis focusing on kinase-coding genes, a prominent category of drug targets, emphasized the importance of identifying and addressing ADRs unique to the Indian population. These insights hold the potential to advance the development of pre-clinical and post-market screening techniques specifically tailored for monitoring ADRs in India (30). Due to the high genetic variability of the populations in lower and middle income countries (LMICs), genotyping is necessary before PGx clinical use (31). However, recent surveys showed that limited PGx research in LMICs has resulted in lower adoption of PGx testing (32).

### PGx research in Qatar and the Middle East region

PGx research in the Middle East has gained prominence due to the region’s distinctive genetic diversity, historical population movements and cultural interactions. This unique genetic makeup has significant implications for drug response variability among individuals. Researchers are actively investigating genetic factors influencing drug metabolism and responses, aiming to personalize treatments, improve drug effectiveness, and reduce adverse reactions. This research has the potential to improve healthcare outcomes, especially in populations with diverse genetic backgrounds like those found in the Middle East. Qatar has been in the forefront of PGx research among the countries in the Middle East region (33). This region is characterized by a high genetic diversity and admixture due to historical migrations and cultural exchanges. Therefore, it is possible that there are unique genetic variants or haplotypes in this region that affect response to drugs such as warfarin. To explore this possibility, a study was conducted on warfarin dosage requirement in patients. The study included 132 Qatari (discovery) and 50 Egyptian (replication) patients who were genotyped using Illumina Multi-Ethnic Global BeadChip Array. This study performed a meta-analysis, combining the Qatari and Egyptian cohorts, and a gene-based analysis to identify genes associated with warfarin dose variability. The results showed that the most significant genetic variants associated with warfarin dose requirements were located in chromosome 16, near the *VKORC1* gene. The lead genome wide signal was *VKORC1* rs9934438 (β = −0.17, *p* = 6 × 10^−15^), which is a well-known variant that has been previously reported in other populations. The study also identified other SNPs in chromosome 10 at a *p* value less than 1 × 10^−5^, but these SNPs did not reach genome wide significance. The genetic variants within *VKORC1* rs9934438 and *CYP2C9* rs4086116 explained 39 and 27% of the variability in the weekly warfarin dose requirement in the Qatari and Egyptians, respectively. These results are consistent with previous studies that have shown that *VKORC1* and *CYP2C9* are the main genetic factors influencing warfarin dose response (34).

Another study was conducted aimed at evaluating the economic benefit of using a genotype-guided approach to determine the optimal number of days to interrupt warfarin before a surgical procedure, compared to the standard of care, in Hamad Medical Corporation (HMC), Qatar. The study used a cost–benefit analysis based on a 1-year decision-analytic model. The study reported that the genotype-guided approach would reduce the risk of bleeding and thrombotic events, as well as the number of canceled procedures, by optimizing the preoperative INR level according to the patient’s genetic variants in *CYP2C9* genes, which affect warfarin sensitivity and metabolism. It was estimated that the genotype-guided approach would result in a benefit to cost ratio of 4.0. On average, the genetic-guided approach resulted in a cost saving of USD 573.72 (QAR 2,094.07) per patient compared to the standard of care. This study provides strong evidence that implementing a pharmacogenetic-guided approach for pre-operative warfarin management is cost-beneficial in the Qatari healthcare setting (35).

One of Qatar’s significant achievements in the field of precision medicine and PGx is the launch of its own large-scale national genome project in 2015, the Qatar Genome Program (QGP). This ambitious project is generating an extensive database combining whole genome sequencing, other omics data, along with phenotypic data, following recruitment and sample collection through the Qatar Biobank (QBB) (36, 37). QBB supports biomedical research in Qatar by collecting and storing biological samples and health data from the Qatari and other resident population. By collecting and analyzing genetic data specific to the Qatari population, researchers can better understand genetic markers that indicate an individual’s risk of developing certain diseases (36). This data provides a valuable genetic reference point for Qataris, enabling earlier diagnoses and tailored management of diseases. Furthermore, it facilitates the development of predictors of response to drugs that account for the unique genetic variations present in the Qatari population (38).

The Qatar Precision Health Institute (QPHI) is a pioneering institution dedicated to advancing precision healthcare practices in Qatar. Established under the auspices of Qatar Foundation, QPHI focuses on research and implementation of precision medicine, aiming to enhance healthcare quality through the comprehensive study of genomics and multi-omics data. Leveraging over a decade’s worth of valuable research and data collection from initiatives like Qatar Biobank and Qatar Genome, QPHI strives to pioneer personalized approaches to prevent and treat health issues. This institute stands at the forefront of empowering communities by facilitating precision health practices, ultimately fostering the development of healthier and more vibrant societies. This transformative initiative embodies a paradigm shift in healthcare, emphasizing individualized treatments and prevention strategies (39).

QGP established a research consortium involving scientists from several institutions in Qatar to address challenging questions in population genomics, and one of the streams of the research was on PGx (6). This led to the publication of one of the most comprehensive studies in the field of PGx in Qatar involving 6,045 whole genomes from the pilot phase of QGP (40). The goal was to understand the distribution of genetic variation affecting drug responses in Qatar and analyzed 2,629 variants across 1,026 genes that impact 559 different drugs or classes of drugs. The key findings indicate a notable divergence in the allele frequencies of 1,320 variants found in 703 genes associated with 299 drugs, in comparison to other global populations. Additionally, the study specifically focused on 15 genes related to 46 drugs with established clinical implementation guidelines, predicting their potential phenotypic impact. On average, individuals in Qatar carry 3.6 actionable genotypes/diplotypes, which influence the use of 13 drugs for which clinical guidelines exist. Almost 99.5% of the individuals possessed at least one clinically actionable genotype/diplotype. One of the significant results from the study was the increased risk of simvastatin-induced myopathy in approximately 32% of Qataris. This prevalence is higher than observed in many other populations. Conversely, fewer Qataris are expected to require dosage adjustments for the immunosuppressant drug tacrolimus, based on their *CYP3A5* genotypes, compared to populations elsewhere. Importantly, the study also revealed distinct distributions of actionable PGx variations within different Qatari subpopulations (40). Furthermore, a focused study on the psychotropic PGx landscape identified that approximately 2 to 51% of the population studied had actionable genetic variants for serotonin reuptake inhibitors. More than half (52%) of Qatari individuals have actionable metabolizer phenotypes related to *CYP2D6*, *CYP2C19* and *CYP2B6* genes, which can influence their response to tricyclic antidepressants. Also, and for antipsychotics, it ranged from 0.1 to 32%, based on genetic variations in *CYP3A4* and *CYP2D6* (41). The results of these studies have profound implications for the preemptive implementation of PGx, not only in Qatar but also in the broader Middle Eastern region. By understanding the unique genetic landscape affecting drug responses in this population, healthcare providers can better tailor medication regimens to individual patients. This personalization of drug prescriptions has the potential to significantly reduce the personal and financial burden associated with drug inefficacy and adverse reactions, ultimately improving patient care and outcomes (40, 41).

Similar efforts from other countries in the region will enhance our knowledge of genetic variants affecting response to drugs and expedite the clinical implementation in the region. A recent study from Saudi Arabia as part of the Saudi Human Genome Project using next generation sequencing data from close to 12,000 participants identified that 99.2% of individuals from the Saudi population carry at least an actionable PGx variant (42). Studies in a healthy Emirati cohort also identified diverse allele frequencies of several pharmacogenetic variants in the UAE (43). Further studies in cardiovascular patients led to the identification of high frequency of patients treated with suboptimal drug regimens, reiterating the need for implementing PGx testing (44). The Emirati Genome Program in the UAE for mapping the genetic diversity of over 400,000 Emirati citizens, will empower healthcare professionals to personalize medical interventions and preventive measures (45).

## Educational activities to support PGx implementation in Qatar

For PGx to advance and be implemented in clinical settings, awareness plays a crucial role. In 2004, the International Society of Pharmacogenomics (ISP) held a Pharmacogenomics Education Forum at Santorini, Greece. The forum participants discussed the importance of pharmacogenomics education and proposed a document of “Background Statement” and “Recommendations and Call for Action” for deans of education at medical, pharmaceutical, and health schools globally (46). Additionally, the CPNDS has educated the public and several healthcare professionals on the use of PGx and trained hundreds of healthcare professionals in this area (12). The University of Florida conducted a survey to determine the impact of PGx on students’ practical knowledge, which revealed that students who had greater PGx training and practical knowledge performed better on the post-course exam than those who lacked these skills (47). A survey in Japan showed that only 0.4% pharmacists were able to ask for PGx testing based on patient’s PGx before prescribing a drug (48). Similar study in Kingdom of Saudi Arabia showed that only 16% of the students could identify drugs that require PGx testing, but 36% students were willing to use PGx testing before prescribing drug (49). Surprisingly, a recent survey in China showed that 99.1% of pharmacists participated in the study knew about PGx testing and 59% had been involved in PGx testing related services (50). Similarly, with increased awareness and support, it is likely that PGx will be widely adopted in clinical practice in India in the future (51).

Despite the low awareness of PGx implementation among physicians and pharmacists in Qatar, they had a positive attitude towards practicing PGx in the clinical setting (52). Qatar, with its commitment to advance the healthcare system, has recognized the importance of PGx implementation to enhance patient care. To support this endeavor, several educational activities have been organized in the country, targeting healthcare providers and students. This section explores the value of these educational initiatives and highlights the significant steps taken towards incorporating PGx into Qatar’s healthcare landscape (53).

### Healthcare provider workshops and courses

Recognizing the need to enhance healthcare providers’ knowledge and understanding of PGx, various institutions in Qatar have organized workshops and courses. These educational activities aim to provide healthcare professionals with the necessary skills to effectively implement PGx in their practice. By staying up-to-date with the latest research and advancements in the field, healthcare providers can make informed decisions when prescribing medications, thereby improving patient outcomes.

These workshops and courses cover a range of topics, including the basics of PGx, resources and evidence, the important pharmacogenes, methods to evaluate PGx evidence and identify important pharmacogenes, PGx guidelines and informatics. Furthermore, genotyping techniques, interpretation of genetic test results, and the clinical application of PGx in different medical specialties are also covered. Moreover, these courses have shed light on how PGx is relevant across various medical specialties, allowing healthcare providers to appreciate its clinical applications in different contexts. To ensure a well-rounded learning experience, these educational activities combine theoretical knowledge with hands-on training, empowering healthcare professionals to gain practical expertise in utilizing PGx tools and resources (54).

### Precision medicine conferences

Hamad Bin Khalifa University (HBKU) hosted the Advances in Precision Medicine (APM2021) conference, with the specific focus on the theme “Epigenetics and Precision Medicine.” This conference showcased the latest and most exciting advancements in the field of epigenetics, especially concerning their implications for clinical decision-making, diagnostic approaches, and treatment strategies (55). Sidra Medicine, along with other institutional partners in Qatar, conducts the annual Precision Medicine and Functional Genomics (PMFG) Symposium, which brings a large number of experts to Qatar. This acts as a recurring platform that brings together researchers, healthcare professionals, policymakers, and community members from around the world. The Hamad Medical Corporation (HMC) has been promoting personalized medicine and PGx implementation, especially in cardiology and oncology. HMC has organized conferences, providing a platform for healthcare professionals, researchers, and stakeholders to share knowledge, experiences, and best practices in the field (56).

Besides this, the World Innovation Summit for Health (WISH) conferences in Qatar have been at the forefront of discussions and advancements in the field of personalized medicine. WISH is a global initiative that convenes healthcare professionals, policymakers, researchers, and experts from various disciplines to address pressing healthcare challenges and drive innovation in healthcare delivery. WISH conferences have highlighted the following key aspects related to personalized medicine: Genomic Medicine: the importance of genomics and genetic testing in understanding disease susceptibility and designing targeted therapies. The conferences have showcased breakthroughs in genomics research and their potential applications in clinical practice. Patient-Centered Care: personalized medicine places patients at the center of their healthcare journey. Discussion on strategies for involving patients in decision-making, ensuring their preferences are considered, and improving overall healthcare outcomes happens during the conference. Data and Technology: the conferences have explored the critical role of data analytics, artificial intelligence, and digital health technologies in advancing personalized medicine. Global Collaborations: it has encouraged international collaboration and partnerships to advance research and implementation of personalized medicine. Ethical and Regulatory Considerations: it also delved into the ethical, legal, and regulatory aspects of personalized medicine, addressing issues such as data privacy, consent, and equitable access to advanced treatments (6, 53). These themes are from various WISH conferences and the readers are referred to the reports from WISH conferences over the years for more details: https://wish.org.qa/reports/.

These conferences provide a valuable chance to learn from experts in personalized medicine, stay updated on cutting-edge research and technology, and discuss challenges and solutions. Sharing knowledge and experiences among professionals enhances our understanding of personalized medicine’s potential and fosters collaboration between different disciplines. These interactions contribute to innovative strategies for implementing precision medicine and PGx effectively in Qatar.

### Inclusion of PGx in the university curricula

Recognizing the potential impact of PGx on future healthcare professionals, colleges and universities in Qatar have taken proactive steps to include PGx in their health sciences curricula. By integrating this emerging field into the coursework, students are exposed to the fundamental concepts of PGx, its clinical implications, and the role it plays in personalized medicine. In 2017, Hamad Bin Khalifa University (HBKU), in collaboration with the QGP, launched a comprehensive program offering Master of Science and Ph.D. degrees in Genomics and Precision Medicine. This program not only emphasizes the study of precision medicine through various relevant topics, but also sheds light on the significant role of PGx. A full course on PGx is available to the students, which has a focus on clinical implementation and includes problem-based learning exercises. As research-oriented degrees, these programs equip the students to be aware of various facets of precision medicine, while making them expert in the chosen field of research. Through this initiative, HBKU aims to advance research and education in these fields, raising a deeper understanding of how genomics and precision medicine can revolutionize healthcare practices, including the critical insights provided by PGx (57).

On the other hand, Qatar University is providing a Master of Science program in Genetic Counseling, which trains students who can contribute greatly to precision medicine field through the development of expertise in risk assessment and psychosocial counseling. As genomic medicine generally involves sensitive genetic information, the genetic counselors can assist in the adoption of genetic testing, providing essential psychosocial support to patients and family members, addressing the emotional and psychological aspects of their genetic condition. Given the diverse ethnicities in the Middle East region, understanding unique issues and challenges related to genetics within these populations will help in delivering personalized healthcare. Lastly, it will provide them with ethical principles that can guide genetic counseling practice, such as the interpretation and applying genetic counseling skills in relation to Sharia and local laws (58).

By integrating PGx knowledge into students’ education early on, Qatar is raising a new generation of healthcare professionals who are highly skilled in this field, aiming to facilitate a smooth transition towards personalized medicine approaches.

### Establishment of committees

Development of institutional and national level committees provide momentum to implement precision medicine across the nation. For example, the Hamad Medical Corporation (HMC) has established a precision medicine committee (PMC) responsible for overseeing and advancing precision medicine initiatives. This committee comprises representatives from various healthcare departments within the hospital, including but not limited to cardiology, oncology, and other specialties. One of the primary objectives of this committee is to collaborate with healthcare providers to smoothly incorporate PGx information into the clinical decision-making process (56).

To advance the field of PGx, international collaboration is crucial. Qatar is actively encouraging global engagement with its data, and the findings from the research initiatives are publicly accessible for specialists and researchers worldwide (37, 59). Collaborations with institutions like Genomics England allow for cross-analysis with national datasets, contributing to a broader understanding of genetic variations across populations. QGP and QBB also contributed to the large international consortia on COVID-19 (60) and the Global Biobank Meta-analysis Initiative (GBMI) (61).

As Qatar’s PGx initiatives progress, the hope is to foster greater collaboration among pharmaceutical companies and researchers worldwide. By leveraging international expertise and conducting comparative studies, Qatar aims to advance the implementation of PGx on a global scale. These research efforts will undoubtedly shape the future of personalized medicine and improve healthcare outcomes not only in Qatar but also in Arab populations and beyond.

## Potential challenges and solutions for PGx implementation

### Challenges and barriers

Despite the recent innovations in PGx, the field has yet to be broadly adopted in clinical practice worldwide. There are several reasons for this slow adoption. One of the main obstacles to implementing PGx is the fact that data obtained have limited cost effectiveness (62). The cost of genome sequencing has decreased dramatically in recent years; however, it is still seen as relatively expensive. As such tests are not part of the standard care, it is not expected to be covered by insurance companies. This makes it difficult for some patients to access genetic testing. The implementation of PGx is expensive and difficult to apply even in high income countries. Therefore, health care systems in less developed countries, who are expected to be low income as well, find it even more challenging (63).

The PGx testing results are often complex and can be difficult to interpret. There are minor inter- individual variation in genetic makeup, and it is not something easy to identify. It is very clear that there are several diverse disease alleles that lead to various disorders (allelic heterogeneity), and multiple enzymes are involved in drug metabolism following different pathways (64). The fact that more than a single gene could be involved in a drug mechanism of action makes it challenging for healthcare providers to use the information to guide treatment decisions. Not only that, but the deficiency and inconsistency of guidelines for clinical practice and the very few recommendations on how to incorporate genetic testing into routine care makes it even more difficult for clinicians to interpret genetic test results and to use the information to advise treatment decisions (65).

The fact that majority of PGx studies have been conducted in populations of European descent, may limit the generalizability of the findings of these studies to other populations (66). This highlights the need for diversity in research population and testing. Another obstacle is the insufficiency of validation of study results. Many PGx tests are not standardized. Different labs use disparate methods and report results in different ways (67). Furthermore, patients need to be willing to participate in genomic medicine projects, which can be influenced by public policy debates on social and political barriers. These include concerns about medical ethics, confidentiality of genetic records, and potential abuses of genetic information (68). Finally, there are limited knowledge and awareness regarding PGx among both clinicians and patients. Senior physicians may not have studied PGx in medical school or during their training, thus they lack the familiarity needed to implement it routinely or appropriately advise patients (69). Many healthcare providers may not be familiar with genetic testing and how to use these results to make treatment decisions. Thus, implementing PGx involves several social, ethical and legal challenges, including those related to population structure and religious biases (62, 70). Overcoming these barriers will be essential for the successful integration of PGx and personalized medicine in the clinical field.

### Addressing the challenges and promoting inclusivity in PGx

**Figure 1:**
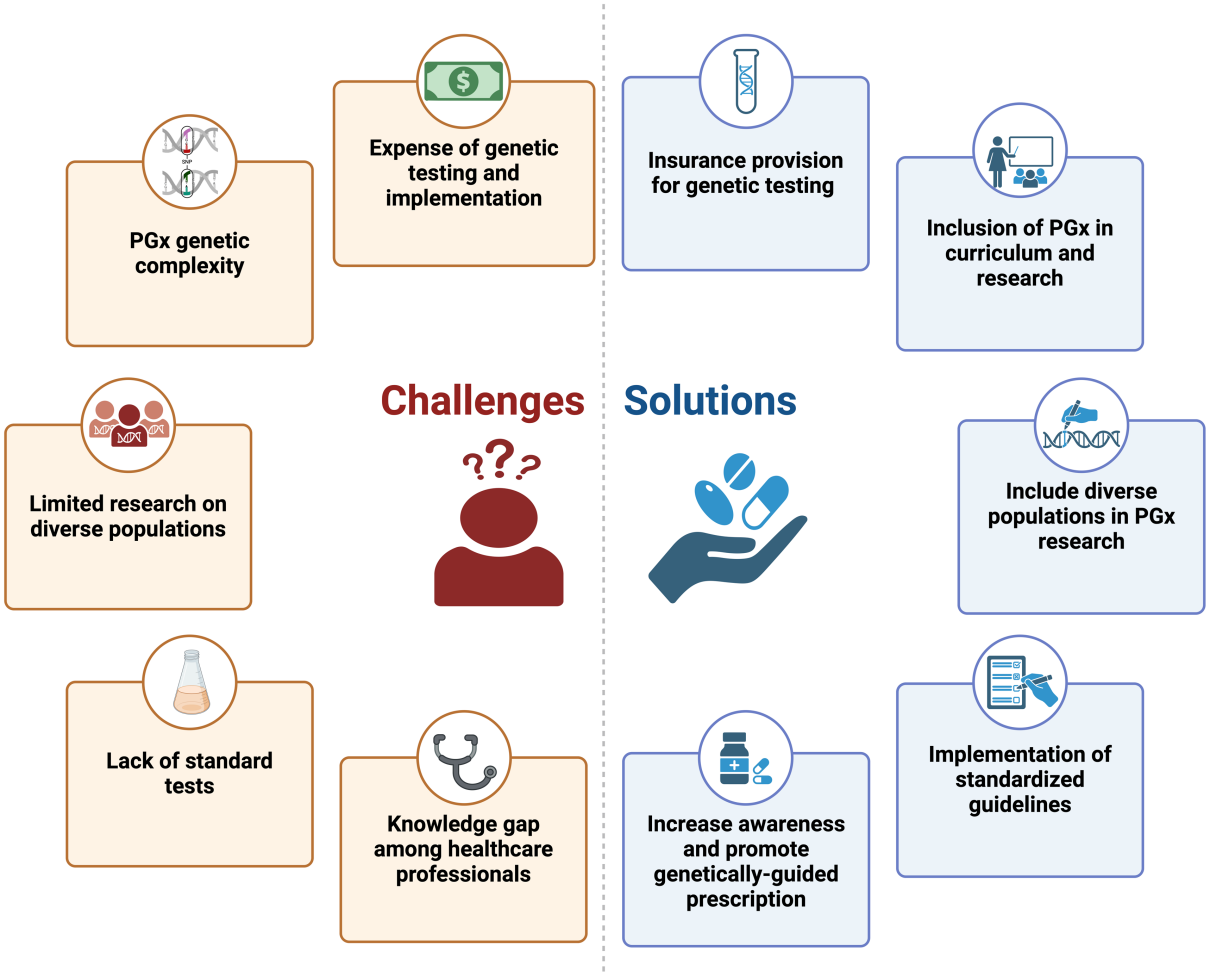
Pharmacogenomics challenges and solutions: an illustrated perspective.

As discussed, PGx holds great promise in advancing precision medicine worldwide, but it faces several challenges, including limited diversity in genomic research and the lack of extensive infrastructure for implementation. Figure 1 shows some of the challenges and potential solutions in PGx implementation. To ensure that progress in PGx benefits all, it’s vital to address the challenges and promote inclusivity in research and clinical settings (71–76).

Sufficient training of Medical Genetics specialists: More specialists must be trained to effectively apply genetic testing results in clinical settings. Infrastructure for medical ethics should be established to incorporate medical ethics into clinical trials that use personalized genetic data. Robust measures are required to ensure the confidentiality and security of genetic records to build public trust. Developing a regulatory oversight infrastructure is crucial to safeguard the public from potential misuse or mishandling of genetic information.

Enhancing diversity in genomic research: To overcome the issue of predominantly studying populations of European ancestry, efforts must be made to include diverse ethnic groups in genomic research. Efforts like the QGP are working towards such inclusivity. Collaborative initiatives like the “All of Us” project in the US, which aims to recruit a million citizens from diverse backgrounds, can be replicated globally. By including individuals from various ethnicities and minorities, researchers can gain a comprehensive understanding of how genetic variations impact drug responses across populations, thus advancing the field of PGx.

Improving infrastructure for PGx implementation: Many healthcare systems lack the infrastructure to implement widespread PGx screening. To overcome this, investments should be made in developing efficient PGx testing processes and integrating genetic data with electronic health records (EHRs). This would allow clinicians to access relevant genetic information at the point of care, facilitating personalized medication decisions and refining prescription guidelines.

Encouraging robust relations between investigators and participants in research: By building trust and collaborative relationships, this can support knowledge exchange, and mutually benefits for both parties. Researchers can share insights associated with PGx findings and its implications. This will empower participants to gain a better understanding of their genetic makeup and potential tailored treatment decisions.

Promoting genetically guided prescription (GGP): As patients’ health outcomes and genetic data both are recorded in the EHRs, researchers can analyze the impact of genetic variations on drug responses/ adverse effects, which will lead to improved medication selections and enhanced overall patient care.

Building mechanistic understanding of PGx: Increasing diversity in both research and clinical settings will enrich the pool of genetic variation data. To unlock the complete potential of PGx, it is vital to tackle the challenges of limited diversity in research and the lack of infrastructure for implementation. By making genomic research more inclusive, strengthening participant-researcher connections, and investing in PGx infrastructure, we can move forward for precise medicine, ensuring that PGx-guided treatments benefit more diverse population.

## Future directions

Over the past several decades, PGx has evolved from an emerging science into a vital interdisciplinary field crucial for personalized medicine. It began as pharmacogenetics, which showed how some genes influence our response to drugs. Randomized Controlled Trials (RCTs) confirmed these genetic discoveries. Advances in genomics has then taken the field to the next level (2, 77).

As we conclude this exploration of the current landscape, it is imperative to consider the future directions and offer recommendations to propel PGx into mainstream clinical practice (2, 72, 74, 78).

### Efforts required worldwide

- Continued investment in research and education: The foundation of successful PGx implementation lies in robust research and education efforts. Governments, research institutions, and pharmaceutical companies should continue to invest in PGx studies to expand our knowledge of genetic variation and drug responses. Concurrently, healthcare professionals should receive training to effectively interpret and apply PGx data in clinical decision-making.
- Ethical considerations and inclusivity: As PGx advances, ethical considerations become more significant. Policy makers, researchers, and healthcare providers should emphasize on the ethical use of genetic information, ensuring privacy and non-discrimination. Additionally, joint efforts should be made to include underrepresented populations in PGx research, to create guidelines that consider genetic diversity.
- Innovations in PGx technology: As technology continues to progress, there will be more efficient and cost-effective methods for conducting PGx testing. Advances in high-throughput sequencing, microarrays, data analysis and computation tools will facilitate widespread implementation of PGx testing, making it more accessible to healthcare providers.
- Integration PGx with EHRs: The integration of PGx data into EHRs will be important for its effective utilization in clinical decision-making. This integration will enable healthcare providers to access and interpret genetic information at the point of care, leading to more precise treatment selection.
- Global Collaborations and Data Sharing: PGx research relies heavily on large-scale datasets to identify significant genetic associations. Global collaborations and data sharing initiatives will become increasingly important to pool resources, accelerate research, and improve the understanding of how genetic variations impact drug responses across diverse populations.
- Conducting standardized randomized clinical trials (RCTs) holds paramount significance in advancing the field of PGx and helping its implementation. While there are contrasting perspectives suggesting potential delays (79), rigorous cross-country RCTs are essential for robust scientific validation and informed decision-making regarding PGx integration into clinical practice. Standardized RCTs are important for several reasons, including that they provide a rigorous and unbiased evaluation of the effectiveness of PGx testing in clinical practice. Also, it helps to identify the most effective testing strategies and inform policy decisions regarding the implementation of PGx in healthcare systems worldwide. Additionally, it addresses concerns about the cost-effectiveness of PGx testing.
- PGx in public health initiatives: PGx can contribute significantly to public health initiatives by optimizing drug treatment in populations with specific genetic characteristics. This approach can help reduce healthcare costs and improve overall health outcomes.
- Cost-Effective implementation: PGx testing should not be cost-prohibitive. Efforts should be directed towards reducing the cost of genetic testing and medications informed by PGx.

### Future directions for PGx in Qatar

In addition to the points mentioned above, here we present some recommendations for Qatar to embrace PGx implementation seamlessly.

- Preemptive PGx testing implementation: Qatar’s vision for the future of PGx involves incorporating preemptive PGx testing into routine clinical practice. By proactively testing individuals for genetic variations relevant to drug response, healthcare providers can make more informed treatment decisions and personalize medication regimens for better patient outcomes.
- Well-designed RCTs to prove the significance of pharmacogenomics: Incorporating RCTs ensures evidence-based practices and strengthens the foundation of PGx implementation in clinical settings. Rigorous experimental designs and concurrent comparison groups are required, as majority of the existing studies were conducted as single arm trials without control groups. Long-term follow-up studies are also required to check whether PGx and personalized medicine improve patient outcomes, adherence, satisfaction, and cost effectiveness. Another requirement is for bigger sample size, which will give sufficient power for the primary outcome. These comprehensive efforts collectively pave the way for a future where precision medicine based on genetic information becomes an integral part of healthcare.
- Patient-centered care model: Qatar’s healthcare transformation efforts emphasize a patient-centered approach to care. PGx aligns perfectly with this model, as it empowers patients to be active participants in their treatment plans, leading to greater patient satisfaction and improved treatment adherence.
- Building infrastructure and informatics capacity: To fully realize the benefits of PGx, Qatar should aim to develop the necessary infrastructure for efficient PGx testing, data storage, and analysis. Building bioinformatics capacity will enable the accurate interpretation of genomic sequences and the production of clinical-grade reports for actionable medications, as recommended by the clinical implementation consortia.
- Collaborations with international PGx initiatives: Qatar’s participation in global PGx collaborations will foster knowledge exchange and ensure that the country remains up-to-date with the latest advancements in the field. Collaborating with international experts will also aid in establishing best practices for PGx testing and implementation.
- Public awareness and education: As PGx becomes an integral part of healthcare in Qatar, public awareness and education will be crucial. Initiatives to inform both healthcare professionals and the general population about the benefits and implications of PGx testing will promote its acceptance and utilization.

## Conclusion

The future of PGx worldwide and in Qatar is promising, with advancements in technology, increased data sharing, and precision drug development driving personalized medicine to new heights. Qatar’s vision to implement preemptive PGx testing aligns perfectly with its patient-centered care model and healthcare reform efforts. By building the necessary infrastructure, bioinformatics capacity, and collaborating with international PGx initiatives, Qatar aims to leverage PGx to optimize medication therapy and improve patient outcomes, as well as decrease side effects.

## Author contributions

KB: Conceptualization, Investigation, Project administration, Supervision, Writing – original draft, Writing – review & editing. DV: Investigation, Methodology, Project administration, Supervision, Writing – original draft, Writing – review & editing. AI: Formal analysis, Investigation, Writing – original draft, Writing – review & editing. MA: Formal analysis, Investigation, Writing – original draft, Writing – review & editing. SM: Investigation, Writing – original draft, Writing – review & editing. HM: Investigation, Writing – original draft, Writing – review & editing. MQ: Investigation, Supervision, Writing – original draft, Writing – review & editing. PJ: Conceptualization, Investigation, Project administration, Supervision, Writing – original draft, Writing – review & editing.
